# Long non‐coding RNA H19 promotes leukocyte inflammation in ischemic stroke by targeting the miR‐29b/C1QTNF6 axis

**DOI:** 10.1111/cns.13829

**Published:** 2022-03-24

**Authors:** Guangwen Li, Xiaoqing Ma, Haiping Zhao, Junfen Fan, Tianwei Liu, Yumin Luo, Yunliang Guo

**Affiliations:** ^1^ 235960 Department of Neurology The Affiliated Hospital of Qingdao University Qingdao China; ^2^ Institute of Integrative Medicine Qingdao University Qingdao China; ^3^ Cerebrovascular Diseases Research Institute and Department of Neurology Xuanwu Hospital of Capital Medical University Beijing China; ^4^ Institute of Cerebrovascular Diseases Medical Research Center the Affiliated Hospital of Qingdao University Qingdao China

**Keywords:** C1QTNF6, inflammation, ischemic stroke, leukocyte

## Abstract

**Aims:**

Inflammatory processes induced by leukocytes are crucially involved in the pathophysiology of acute ischemic stroke. This study aimed to elucidate the inflammatory mechanism of long non‐coding RNA (lncRNA) H19‐mediated regulation of C1q and tumor necrosis factor 6 (C1QTNF6) by sponging miR‐29b in leukocytes during ischemic stroke.

**Methods:**

H19 and miR‐29b expression in leukocytes of patients with ischemic stroke and rats with middle cerebral artery occlusion were measured by real‐time polymerase chain reaction. H19 siRNA and miR‐29b antagomir were used to knock down H19 and miR‐29b, respectively. We performed in vivo and in vitro experiments to determine the impact of H19 and miR‐29b on C1QTNF6 expression in leukocytes after ischemic injury.

**Results:**

H19 and C1QTNF6 upregulation, as well as miR‐29b downregulation, was detected in leukocytes of patients with stroke. Moreover, miR‐29b could bind C1QTNF6 mRNA and repress its expression, while H19 could sponge miR‐29b to maintain C1QTNF6 expression. C1QTNF6 overexpression promoted the release of IL‐1β and TNF‐α in leukocytes, further exacerbated blood‐brain barrier disruption, and aggravated the cerebral ischemic injury.

**Conclusions:**

Our findings confirm that H19 promotes leukocyte inflammation by targeting the miR‐29b/C1QTNF6 axis in cerebral ischemic injury.

## Graphical abstract

Schematic diagram depicting the possible involvement of H19/miR‐29b/C1QTNF6 axis in cerebral ischemic stroke. Ischemia‐dependent activation of the H19/miR‐29b/C1QTNF6 axis enhances IL‐1β and TNF‐α expression in leukocytes; moreover, it causes further BBB disruption to aggravate neuronal injury.

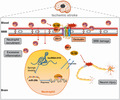

## 1 INTRODUCTION

Stroke is a major cause of mortality and morbidity worldwide.^1^, ^2^ Ischemic stroke, caused by thrombosis or embolism in the blood vessels, is the most common subtype of stroke.^3^ Currently, there is no specific treatment to improve the prognosis of patients with ischemic stroke due to its complex pathogenesis. A series of critical post‐stroke inflammatory responses are involved in functional recovery after ischemic injury. These inflammatory responses further determine the prognosis of stroke.

Peripheral leukocytes significantly affect the clinical outcome of stroke and can be a therapeutic target to treat and prevent ischemic stroke.^4^, ^5^, ^6^ There have been several studies on the expression of non‐coding ribonucleic acids (ncRNAs) in peripheral leukocytes of patients with ischemic stroke. The ncRNAs in leukocytes regulate the release of inflammatory factors to promote inflammatory responses after cerebral ischemic injury.^7^ They can be classified as short (≤200 base pairs) or long ncRNAs (lncRNAs; >200 base pairs).^8^ MicroRNAs (miRNAs or miRs) are ncRNAs with <22 nucleotides that modulate protein expression at the post‐transcriptional level.^4^ miRNAs prevent messenger RNA (mRNA) translation by binding to the consensus seed sequences in the 3′ untranslated regions (UTRs) of target mRNAs.^4^ Recent studies have demonstrated mutual regulation between miRNAs and lncRNAs, including a direct interaction and an indirect role by binding to target genes in ischemic stroke.^9^

To identify the leukocyte genes related to ischemic stroke, we previously analyzed mRNA expression profiles of neutrophils using RNA‐seq and compared the profiles of patients with acute ischemic stroke and healthy controls. We found an alteration in the expression of C1q and tumor necrosis factor 6 (C1QTNF6) after ischemic stroke. C1QTNF6 was originally identified in the heart, brain, and peripheral inflammatory cells. It is known to be involved in diverse processes, including apoptosis, metabolism, and release of inflammatory factors.^10^ C1QTNF6 can directly modulate pro‐inflammatory cytokine gene expression in leukocytes.^11^ Our preliminary findings demonstrated that C1QTNF6 mRNA could be modulated by several miRNAs in neutrophils, including miR‐29b. The expression of miR‐29b in leukocytes is significantly decreased after ischemic injury, and changes in the miR‐29b level are correlated with the prognosis of ischemic stroke.^12^ In contrast, miR‐29b overexpression alleviates brain injury and attenuates blood–brain barrier (BBB) damage.^12^ We predicted the potential targeting lncRNAs related to miR‐29b and identified the binding site of miR‐29b in H19 using StarBase. According to a report, H19 levels increase in the circulating blood after ischemic stroke, which promotes the release of inflammatory factors.^13^ Therefore, we hypothesized that C1QTNF6 in leukocytes might be regulated by H19 and miR‐29b that are probably involved in the pathogenesis of ischemic stroke.

This study aimed to elucidate the inflammatory mechanism of lncRNA H19‐mediated regulation of C1QTNF6 by sponging miR‐29b in leukocytes during ischemic stroke.

## 2 MATERIALS AND METHODS

### 2.1 Participants

This study was registered with ClinicalTrials.gov (NCT03577093). We recruited 50 patients with acute ischemic stroke of anterior circulation from the emergency department of Xuanwu Hospital between March 2016 and December 2017. Ischemic stroke was diagnosed by two neurologists based on the patient history, laboratory examinations, neurological deficits, and diffusion‐weighted magnetic resonance imaging (MRI) findings. We included patients who had ischemic stroke for the first time, aged 18–80 years, had a National Institute of Health Stroke Scale (NIHSS) score <25 points and were admitted within 6 h after the onset of symptoms. For the control group, we included 42 age‐ and sex‐matched healthy volunteers from the medical examination center who did not have any central nervous system disease.

### 2.2 Blood collection and separation of neutrophils

Venous blood samples were collected from patients with ischemic stroke and healthy controls in vacuum tubes containing ethylene diamine tetra‐acetic acid and anticoagulant upon admission. Blood samples were fractionated by centrifugation at 3000 *g* for 10 min at 4°C. Subsequently, the plasma layer was aliquoted and stored at −80°C for routine laboratory assays. Neutrophils were separated using a standard Ficoll‐Paque Plus gradient method for RNA extraction and examined as described in a previous report.^4^

### 2.3 Animals

All experimental protocols were approved by the Institutional Animal Care and Use Committee of the Qingdao University. Animal studies are conducted in compliance with the ARRIVE guidelines 2.0.^14^ Male Sprague–Dawley rats weighing 260–280 g were purchased from Vital River Laboratory Animal Technology Co. Ltd. The animals were maintained in standard‐housing and open‐top cages in a pathogen‐free facility at 23 ± 1°C with 50%–60% humidity at Qingdao University. All animals underwent fasting for 24 h with free access to tap water before the experimental procedures.

### 2.4 Middle cerebral artery occlusion

Rats underwent surgery for right middle cerebral artery occlusion (MCAO). After anesthetization with enflurane, a nylon filament with a silicon tip of 0.37 mm diameter was inserted into the right middle cerebral artery to obstruct blood flow for 2 h. Subsequently, it was removed to allow reperfusion. Regional cerebral blood flow was observed using transcranial laser Doppler to confirm a decrease in blood flow by 20%–30%, compared with the baseline value during MCAO. Sham‐operated rats underwent the same anesthetic and surgical procedures without MCAO.

Rats were randomly divided into six groups: sham, MCAO, MCAO+miR‐29b antagomir, MCAO+H19 siRNA, MCAO+miR‐29b antagomir+H19 siRNA control, and MCAO+miR‐29b antagomir+H19 siRNA. H19 siRNA, miR‐29b antagomir, and transfection reagent were purchased from GenePharma. Three days before the MCAO surgery, the rats were treated with 100 μl of miR‐29b antagomir, H19 siRNA, or control mixed with 32 μl of transfection reagent through the tail vein. The RNA sequences were as follows: 5′‐CCACCGUAAUUCAUUUAGATT‐3′ (H19 siRNA), 5′‐AACACUGAUUUCAAAUGGUGCUA‐3′ (miR‐29b antagomir), and 5′‐UUCUCC GAACGUGUCACGUTT‐3′ (H19 siRNA control).

### 2.5 Behavioral tests

Behavioral tests were performed using 12 animals in each group. Rats underwent neurological evaluation using a Zea‐Longa 5‐point scoring system before and 24 h after surgery.^15^

### 2.6 2,3,5‐Triphenyl‐2H‐tetrazolium chloride (TTC) staining

Rats were sacrificed 24 h after reperfusion. The brains were quickly removed and cut into 2 mm thick coronal slices for TTC staining. The infarct volume was calculated as follows:

$$\begin{array}{c} \left(\text{contralateral}\;\text{hemisphere}\;\text{volume ‐ undamaged}\;\text{ipsilateral}\;\text{hemisphere}\right)\\ \text{/contralateral}\;\text{hemisphere}\;\text{volume}\times 100\text{\%}\;. \end{array}$$

Volume calculation with edema correction was as follows:

$$\begin{array}{c} \left(\text{ipsilateral}\;\text{hemisphere}\;\text{volume ‐ contralateral}\;\text{hemisphere}\;\text{volume}\right)\\ \text{/contralateral}\;\text{hemisphere}\;\text{volume}\times 100\text{\%}\;. \end{array}$$

### 2.7 Western blotting

The ipsilateral cerebral hemisphere, rat leukocytes, and cultured cells were homogenized in a lysis buffer containing phosphatase and protease inhibitors. The venous blood samples were obtained 24 h after MCAO surgery, and the leukocytes were separated as previously described. We used the following primary antibodies: anti‐interleukin 1 beta (IL‐1β) (Catalogue, AF‐501‐NA), anti‐tumor necrosis factor‐alpha (TNF‐α) (Abcam, ab205587), anti‐matrix metallopeptidase 9 (MMP‐9) (Abcam, ab76003), anti‐zonula occludens‐1 (ZO‐1) (Arigo, ARG55738), anti‐occludin (Abcam, ab216327), anti‐caspase‐3 (Abcam, ab13847; Affinity Biosciences, AF7022), anti‐C1QTNF6 (Abcam, ab36900), and anti‐β‐actin (Abcam, ab20272). Proteins were detected by incubation with horseradish peroxidase‐conjugated secondary antibodies (Santa Cruz Biotechnology) for 60 min at room temperature, using an enhanced chemiluminescence kit (Millipore). Gray values of the protein bands were analyzed using AlphaEase FC software (Alpha Innotech).

### 2.8 Real‐time polymerase chain reaction (RT‐PCR)

Equal quantity of purified RNA was obtained from neutrophils, leukocytes, and brain tissues and used as a template for cDNA synthesis using SYBR Green qPCR Master Mix (Fermentas), oligo‐d(T) primers, and SuperScript III/RNaseOUT Enzyme Mix (Invitrogen). miR‐29b primer sequences for human neutrophils were 5′‐GGGTAGCACCATTTGAAATCA‐3′ and 5′‐GTGCGTGTCGTGGAGTCG‐3′. MiR‐29b primer sequences for rat leukocytes were 5′‐CTCAACTGGTGTCGTGGAGTCGGCAATTCAGTTGAG‐3′ and 5′‐ACACTCCAGCTGGGTAGCACCATTTGAAATC‐3′. H19 primer sequences for human neutrophils were 5′‐CTTCTTTAAGTCCGTCTCGTTC‐3′ and 5′‐GAGGCAGGTAGTGTAGTGGTTC‐3′. H19 primer sequences for rats 5′‐GGAATCGGCTCGAAGGTAAA‐3′ and 5′‐GGGCCAGGCAGAGTTAGTTG‐3′. C1QTNF6 primers for human neutrophils were 5′‐TCAGTCCCTTCCACCAAA‐3′ and 5′‐ACCTTGATAAAGCCTGGAGA‐3′. C1QTNF6 primer sequences for rats were 5′‐GTTCGGGGTCTGTGAGTTGAG‐3′ and 5′‐CTTTCAGGATGGTGATGTTGATGTA‐3′. The relative gene expression was calculated using the 2^−ΔΔCT^ method, normalized, and expressed as fold change relative to U6.

### 2.9 NeuN/TUNEL staining and immunofluorescence analysis

Rats were killed through chloral hydrate administration, and cardiac perfusion was performed with physiological saline and 4% paraformaldehyde. The brain was harvested and fixed in 4% paraformaldehyde for 48 h, followed by dehydration in 30% sucrose. A cryostat vibratome was used to obtain 20‐μm‐thick coronal sections of the brain. The sections were blocked with 0.3% (w/v) bovine serum albumin in phosphate‐buffered saline (PBS) at room temperature for 1 h and then incubated with NeuN antibodies (Millipore, 1:1000) overnight at 4°C. The slides were washed in Tris‐buffered saline with 0.1% Tween 20, incubated with Alexa Fluor 488 AffiniPure Donkey antibody, and counterstained with DAPI. The number of NeuN/TUNEL‐positive cells in the ischemic region was calculated using ImageJ software (National Institutes of Health, Bethesda, USA). Fluorescence images were acquired using an Olympus Fluoview FV1000 fluorescence microscope (Olympus).

### 2.10 ELISA

Plasma samples were collected from stroke patients and healthy volunteers as previously described.^4^ Approximately 200 μl of plasma was prepared in an ice‐cold PBS. TNF‐α, IL‐1β, and MMP‐9 protein levels were quantified using a commercial ELISA kit (Abcam), following the instructions of the manufacturer.

### 2.11 Cell culture, transfection, oxygen‐glucose deprivation treatment, and co‐culturing

The human acute myeloid leukemia cell line, HL‐60, was used as a neutrophil model. HL‐60 cells were cultured in 1640 RPMI medium containing 10% heat‐inactivated fetal bovine serum, 100 U/ml penicillin, and 100 mg/ml streptomycin at 37°C in an atmosphere of 5% CO_2_. HL‐60 cells were transfected with a mixture of H19 siRNA, miR‐29b antagomir or control, and Lipofectamine RNAiMAX (GenePharma). The cells were incubated for 24 h in a humidified incubator for further analyses.

Human brain capillary endothelial cells (hCMEC/D3) were grown in EBM‐2 medium supplemented with vascular endothelial growth factor, insulin‐like growth factor‐1, epidermal growth factor, basic fibroblast growth factor, hydrocortisone, ascorbate, penicillin‐streptomycin, and 2.5% fetal calf serum. The cells were maintained in an incubator at 37°C and 5% CO_2_. Oxygen–glucose deprivation (OGD) and reoxygenation were used as in vitro models for mimicking cerebral ischemia. Cultured hCMEC/D3 cells were kept in a glucose‐free and hypoxic incubator chamber with a gas mixture of 94.5% N_2_, 0.5% O_2_, and 5% CO_2_ at 37°C for 2.5 h, followed by co‐culturing with HL‐60 cells for 24 h. The hCMEC/D3 cells were divided into five groups: Sham (+HL‐60 vehicle‐siRNA), OGD (+HL‐60 vehicle‐siRNA), OGD (+HL‐60 miR‐29b antagomir), OGD (+HL‐60 H19 siRNA), and OGD (+miR‐29b antagomir+H19 siRNA).

In the co‐culture experiments, 10 × 10^5^ hCMEC/D3 cells/well were co‐cultivated with HL‐60 cells treated with H19 siRNAs, miR‐29b, or controls under non‐contact conditions. Furthermore, 10 × 10^5^ HL‐60 cells/well were seeded on the apical side of Transwell membranes (Corning, 0.4 µm pore size) and cultivated with the supplemented medium. Moreover, hCMEC/D3 cells were seeded on the basal side and cultivated in a static monoculture model. After co‐culturing for 24 h, the cells were separately collected for further analysis.

### 2.12 Flow cytometry

The apoptosis ratio was estimated using the Dead Cell Apoptosis Kit with Annexin V Alexa Fluor 488 for flow cytometry, following the instructions of the manufacturer (Invitrogen). Cells were collected, washed twice with cold PBS, and incubated with Annexin V‐FITC mixed with propidium iodide for 10 min in the dark. Cellular fluorescence was assessed using flow cytometry (CytoFLEX S, Beckman).

### 2.13 Statistical analysis

Statistical analyses were performed using SPSS version 23.0. Shapiro–Wilk test to test the normality of the data. Normally distributed data are expressed as mean ± SD. Student's *t*‐test was used for between‐group comparisons. One‐way analysis of variance with the Tukey–Kramer post hoc test was used to compare several quantitative variables. Non‐normally distributed data were evaluated using the Mann–Whitney *U*‐test and were expressed as medians. Pearson's correlation test was used to assess between‐variable correlations. Statistical significance was set at *p* < 0.05.

## 3 RESULTS

### 3.1 Upregulation of H19 and C1QTNF6 and downregulation of miR‐29b in neutrophils of patients with ischemic stroke

**FIGURE 1:**
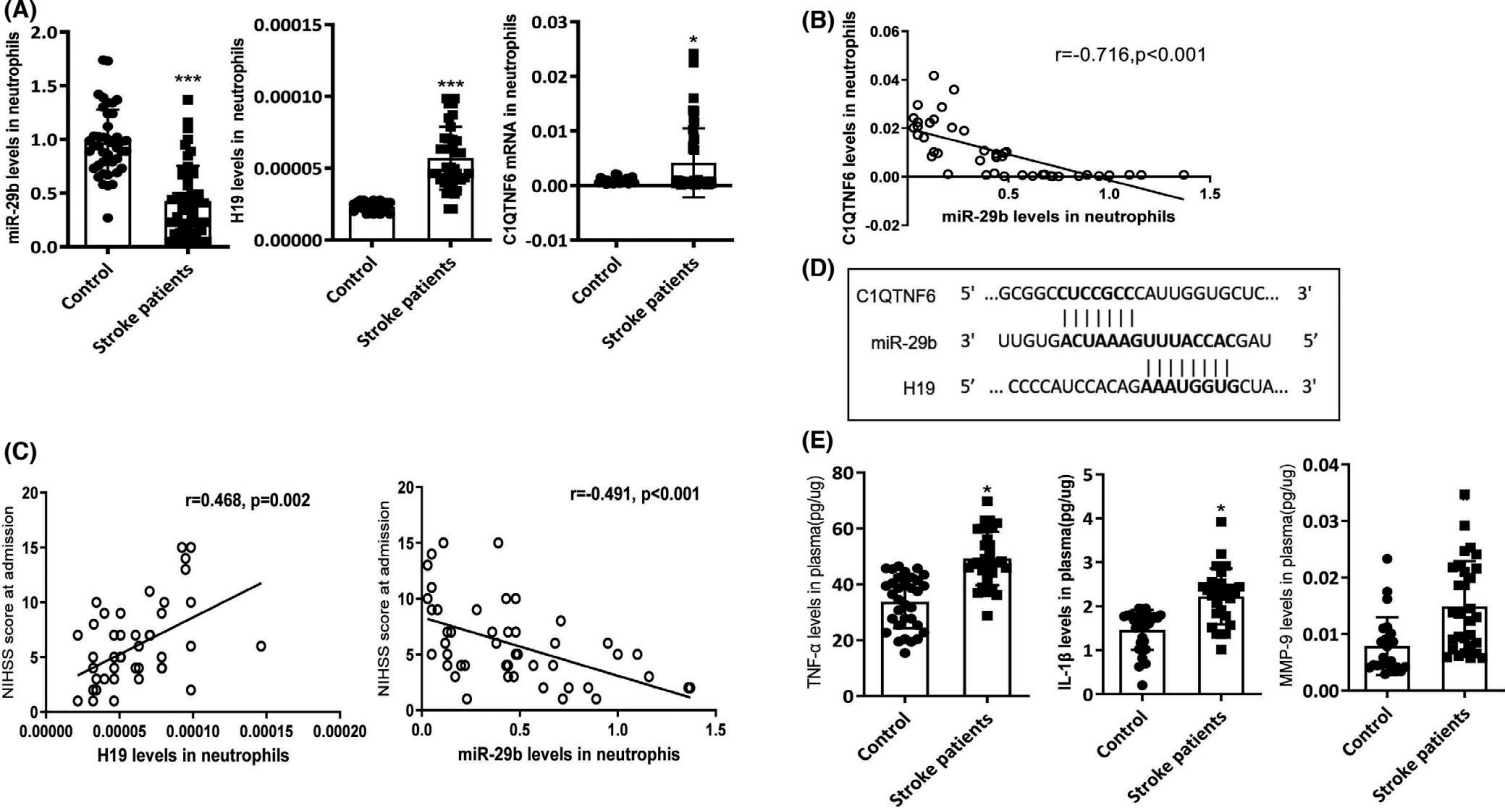
H19, miR‐29b, and C1QTNF6 expression in patients with acute stroke. (A) Quantitative RT‐PCR results for H19, miR‐29b, and C1QTNF6 levels in neutrophils of patients with acute stroke. Acute ischemic stroke group (*N* = 50) and control group (*N* = 42). (B) Correlation between miR‐29b and C1QTNF6 expression levels at admission (*N* = 50). (C) Correlation of H19 and miR‐29b expression with NIHSS score at admission (*N* = 50). (D) The predicted binding sites between miR‐29b and C1QTNF6 and between miR‐29b and H19. (E) Plasma TNF‐α, IL‐1β, and MMP‐9 levels in patients with acute ischemic stroke (*N* = 50). *p < 0.05, ***p < 0.001 vs. control group

We investigated a novel gene, C1QTNF6, which demonstrated increased mRNA levels in neutrophils of patients with acute ischemic stroke compared with healthy volunteers (Figure 1A, *p* < 0.05). Further analysis using TargetScan and StarBase databases revealed that C1QTNF6 is regulated by miR‐29b and H19. We identified the putative miR‐29b binding sites of H19 and 3′ UTR of C1QTNF6 mRNA (Figure 1D). To confirm this, we conducted quantitative RT‐PCR to evaluate mRNA expression levels of miR‐29b, H19, and C1QTNF6 in neutrophils. H19 expression in neutrophils was significantly increased in patients with stroke (Figure 1A, *p* < 0.05). Additionally, the patients had decreased miR‐29b expression (Figure 1A, *p* < 0.05), which significantly increased the C1QTNF6 levels. There was a negative linear correlation between miR‐29b and C1QTNF6 levels in the neutrophils of patients with stroke (Figure 1B, *r* = −0.716, *p* < 0.001).

### 3.2 H19 and miR‐29b levels correlate with the NIHSS score

To further elucidate the clinical significance of H19 and miR‐29b in patients with stroke, we analyzed the correlation of clinical parameters with H19 and miR‐29b expression. H19 levels positively correlated with the NIHSS score at admission (Figure 1C, *r* = 0.468, *p* = 0.002), while miR‐29b levels negatively correlated with the NIHSS score (Figure 1B; *r* = −0.491, *p* < 0.001). Plasma IL‐1β, TNF‐α, and MMP‐9 levels were significantly higher in the patients than in the controls (Figure 1E, *p* < 0.05). These results suggest that H19 and miR‐29b in neutrophils are critically involved in ischemic stroke.

### 3.3 H19 expression is upregulated after MCAO injury, while H19 inhibition alleviates MCAO injury in rats

**FIGURE 2:**
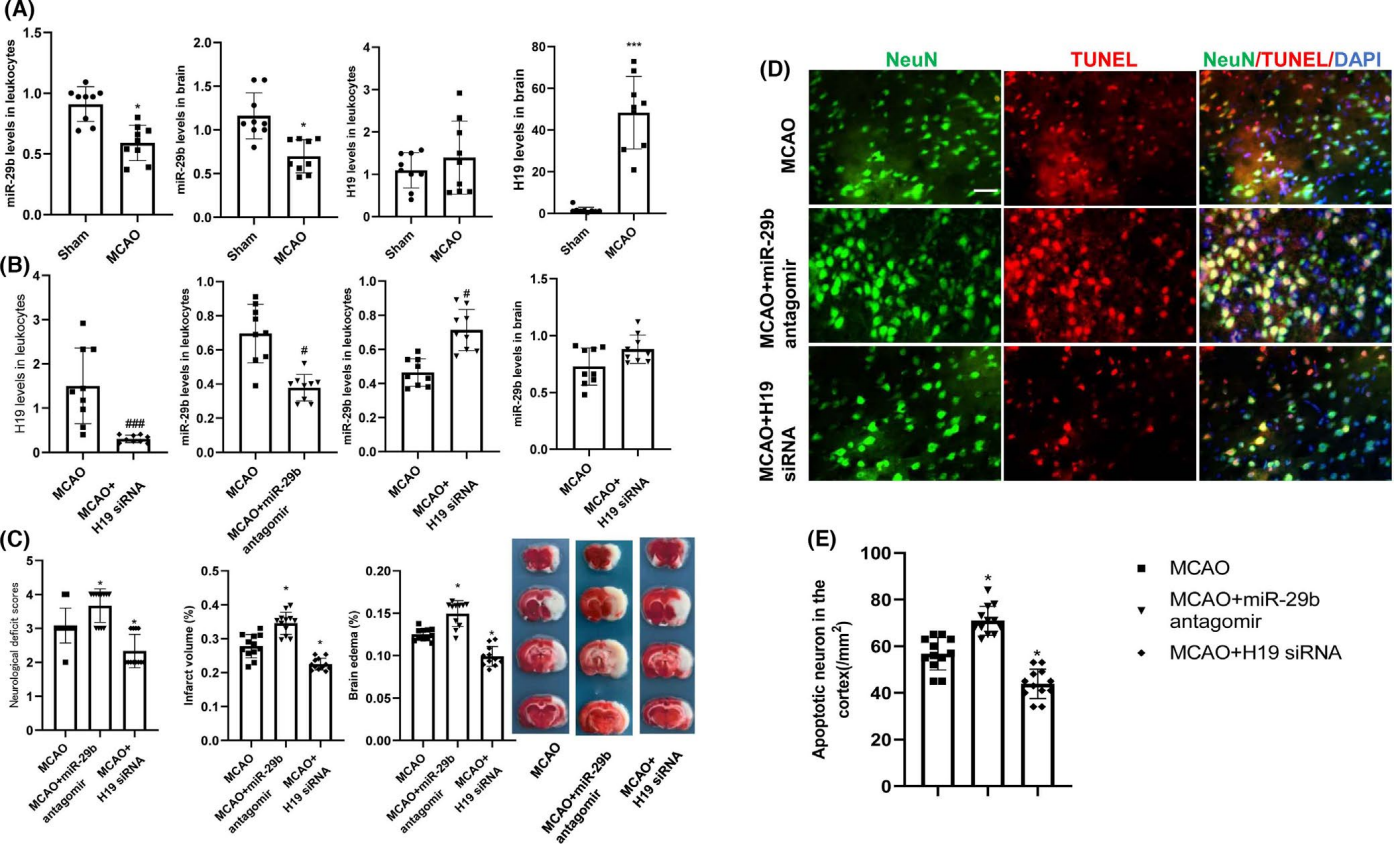
Changes in H19 and miR‐29b levels and subsequent effects on cerebral injury and neurological deficits in MCAO rats. (A) H19 and miR‐29b expression levels in leukocytes and ischemic brain tissues after MCAO injury (*N* = 9). (B) Quantitative RT‐PCR experiment to confirm the efficacy of H19 siRNA and miR‐29b antagomir transfection (*N* = 9). (C) Effects of intravenous injections of H19 siRNA and miR‐29b antagomir on cerebral injury and neurological function deficits at 24 h after stroke (*N* = 12). (D and E) Neuronal apoptosis in the ipsilateral cortex as detected by NeuN/TUNEL immunofluorescence double staining (*N* = 6). *p < 0.01, ***p < 0.001 vs. sham group. ^#^ *p* < 0.05, ^###^ *p* < 0.001 vs. MCAO group. Scale bar = 20 μm

Considering the increased expression of H19 in patients with stroke, we further evaluated the role of H19 in cerebral ischemic injury in rats by intravenous injection of H19 siRNA three days before MCAO surgery. Quantitative RT‐PCR was performed to detect H19 expression and to verify the efficacy of H19 siRNA transfection. Initially, the level of H19 in the ischemic brain of rats was increased 24 h after MCAO injury (Figure 2A, *p* < 0.001). There was no significant difference in the leukocyte H19 levels between MCAO and sham rats; however, there was a trend of upregulation of H19 levels (Figure 2A, *p* > 0.05). H19 expression in the leukocytes decreased after treatment with H19 siRNA (Figure 2B, *p* < 0.001). TTC staining showed that H19 siRNA treatment decreased the cerebral infarct and edema volume compared with those in MCAO rats (Figure 2C, *p* < 0.05). Moreover, H19 siRNA alleviated the MCAO‐induced neurological deficits (Figure 2C, *p* < 0.05).

To determine whether H19 siRNA preserves neurons from ischemic injury, we examined the number of NeuN/TUNEL‐positive cells in rats after MCAO injury. We observed that MCAO injury increased the number of cortical TUNEL‐positive cells as compared to that in sham‐operated rats, which was abrogated by H19 siRNA treatment (Figure 2D,E; *p* < 0.05). These results demonstrate that H19 inhibition can attenuate cerebral ischemic injury in the cortex.

### 3.4 MiR‐29b expression is downregulated after MCAO injury, and miR‐29b inhibition exacerbates cerebral injury

We investigated the effect of intravenous injection of miR‐29b antagomir by measuring the infarct volume and neurological deficits after MCAO injury. First, we found decreased levels of miR‐29b in the ischemic brain and leukocytes in MCAO rats at 24 h after the injury (Figure 2A, *p* < 0.05). Second, there was downregulation of miR‐29b expression in leukocytes after miR‐29b antagomir injection (Figure 2B, *p* < 0.05). Third, TTC staining showed that miR‐29b antagomir treatment increased the cerebral infarct volume and edema volume compared with that in the MCAO rats (Figure 2C, *p* < 0.05). Furthermore, MiR‐29b antagomir significantly promoted MCAO‐induced neurological deficits (Figure 2C, *p* < 0.05). Fourth, miR‐29b antagomir increased the number of cortical TUNEL‐positive cells in MCAO rats (Figure 2D, *p* < 0.05). Lastly, H19 siRNA promoted miR‐29b expression, which indicates that H19 interacts with miR‐29b in the leukocytes of MCAO rats (Figure 2B, *p* < 0.05).

### 3.5 H19 and miR‐29b regulate MCAO‐induced C1QTNF6, IL‐1β, and TNF‐α expression

**FIGURE 3:**
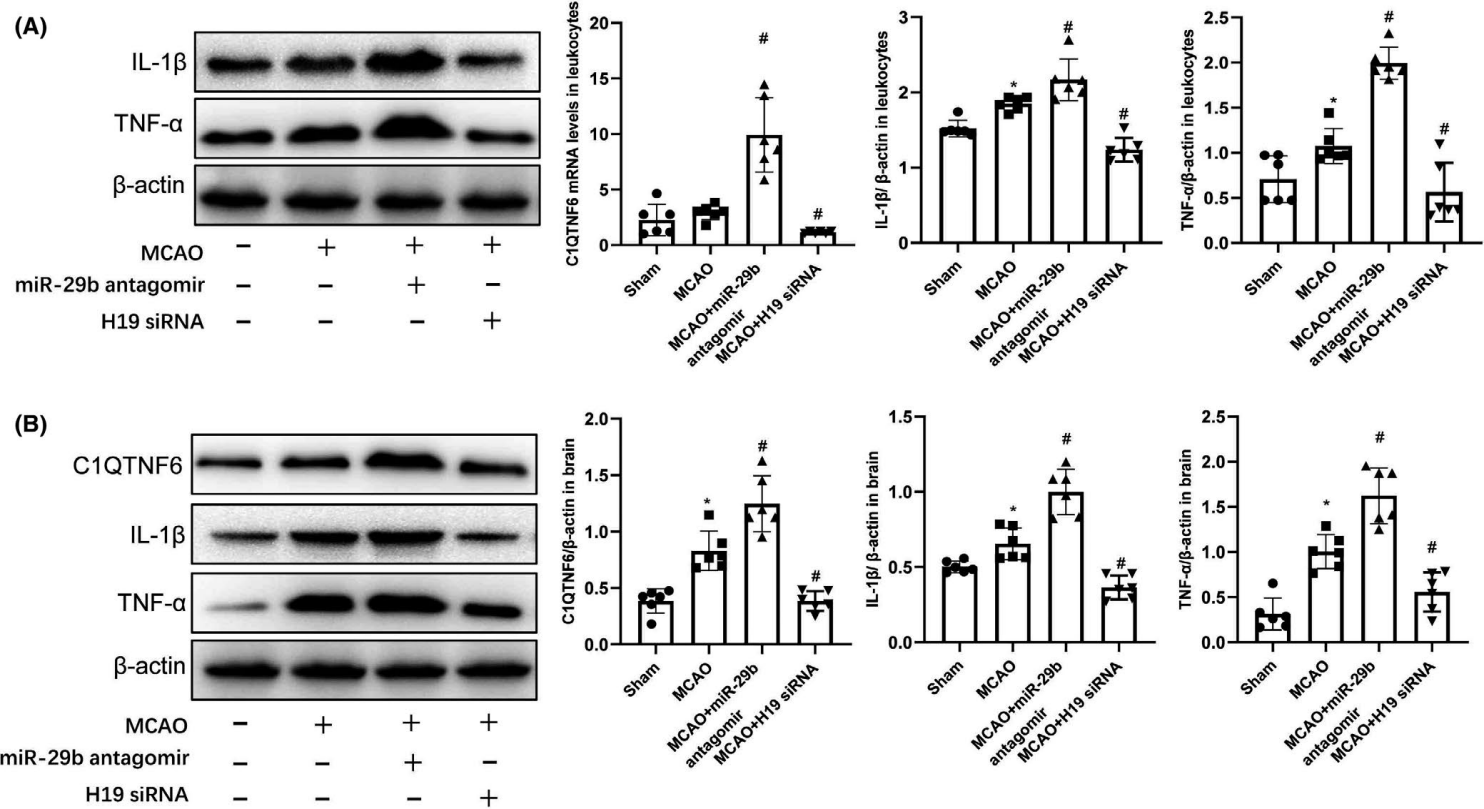
H19 and miR‐29b regulate the expression of C1QTNF6 in the leukocytes of MCAO rats. (A) Leukocyte C1QTNF6, IL‐1β, and TNF‐α protein levels assessed by Western blotting (*N* = 6). (B) Ipsilateral brain tissue C1QTNF6, IL‐1β, and TNF‐α protein levels assessed by Western blotting (*N* = 6). *p < 0.05 vs. sham group; ^#^ *p* < 0.05, vs. MCAO group

To determine whether miR‐29b is involved in the pathophysiology of cerebral ischemic stroke, MCAO rats were intravenously injected with miR‐29b antagomir to decrease miR‐29b expression in leukocytes. First, miR‐29b antagomir treatment significantly increased C1QTNF6 mRNA levels in leukocytes of MCAO rats (Figure 3A, *p* < 0.05). Second, miR‐29b antagomir treatment increased C1QTNF6 protein levels in the brain (Figure 3B, *p* < 0.05). Treatment with miR‐29b antagomir treatment also increased IL‐1β and TNF‐α levels in MCAO rats (Figure 3A,B, *p* < 0.05).

We further analyzed IL‐1β and TNF‐α expression in MCAO rats treated with H19 siRNA. Treatment with H19 siRNA significantly decreased brain and leukocyte levels of IL‐1β and TNF‐α (Figure 3A,B; *p* < 0.05). Moreover, C1QTNF6 mRNA and protein expression in leukocytes and the brain were significantly downregulated in MCAO rats treated with H19 siRNA (Figure 3A,B; *p* < 0.05). These findings suggest that H19 and miR‐29b regulate C1QTNF6 levels and affect IL‐1β and TNF‐α expression in the leukocytes and brains of MCAO rats.

### 3.6 H19 inhibition reverses MCAO injury, which is worsened by miR‐29b antagomir

**FIGURE 4:**
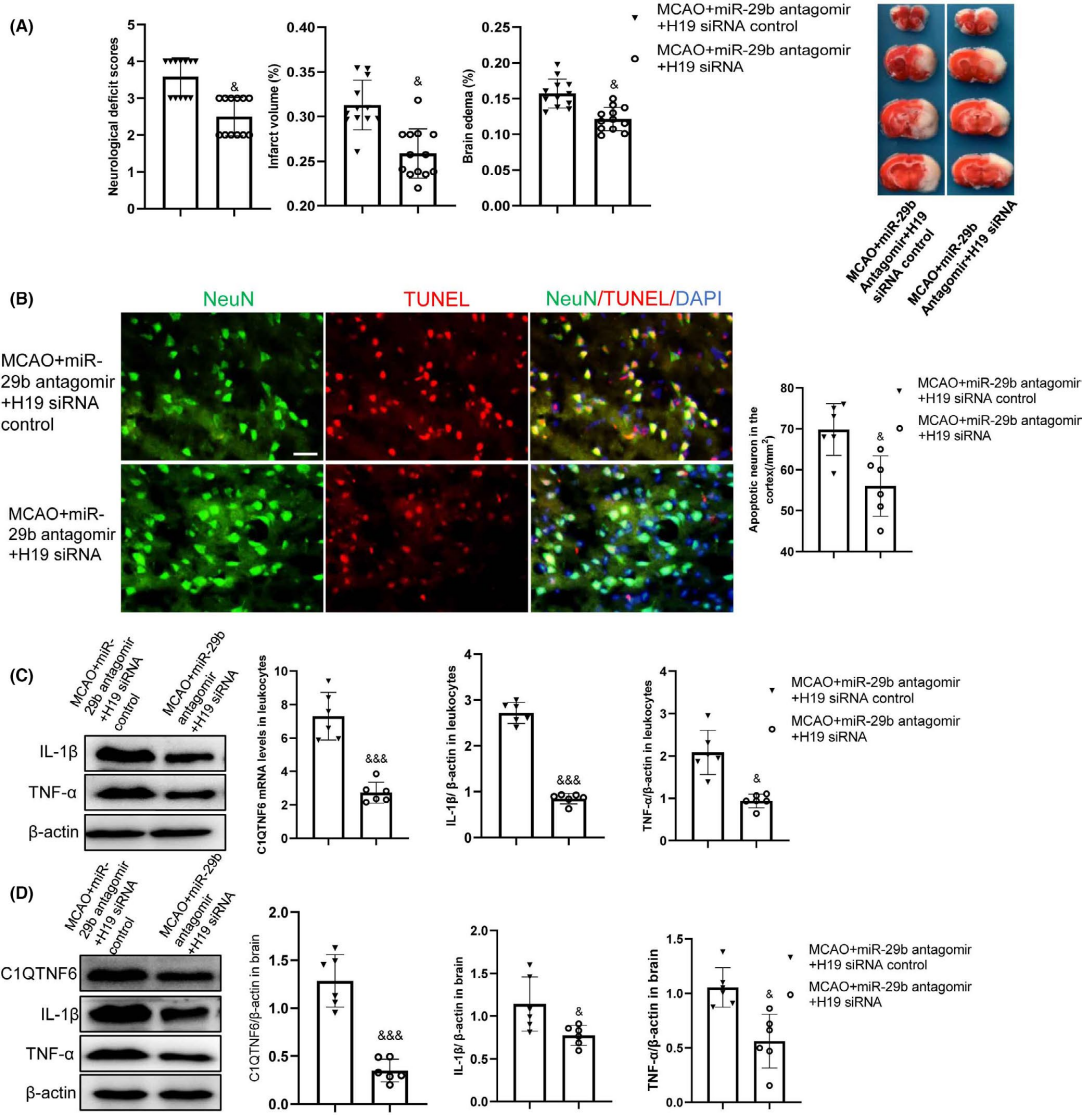
H19 siRNA reverses the miR‐29b antagomir‐aggravated acute cerebral ischemic injury. (A) Cerebral infarct volume and brain edema evaluated by TTC staining of coronal brain sections (*N* = 12). (B) Neuronal apoptosis in the ipsilateral cortex as detected by NeuN/TUNEL immunofluorescence double staining (*N* = 6). (C) Leukocyte C1QTNF6 mRNA, IL‐1β, and TNF‐α protein levels assessed by quantitative RT‐PCR and Western blotting (*N* = 6). (D) Ipsilateral brain tissue C1QTNF6, IL‐1β, and TNF‐α protein levels assessed by Western blotting (*N* = 6). ^&^ *p* < 0.05, ^&&&^ *p* < 0.001 vs. MCAO+miR‐29b antagomir+H19 siRNA control group. Scale bar = 20 μm

To further elucidate the relationship among H19, miR‐29b, and C1QTNF6 in cerebral ischemic injury, we intravenously injected H19 siRNA in MCAO rats with miR‐29b antagomir and analyzed the infarct volume and brain edema at 24 h after MCAO injury. H19 siRNA reduced the cerebral infarct volume and brain edema compared with that in MCAO rats treated with miR‐29b antagomir (Figure 4A, *p* < 0.05). Additionally, H19 siRNA significantly improved the neurological deficits in MCAO rats after miR‐29b antagomir treatment (Figure 4A, *p* < 0.05).

H19 siRNA decreased the number of cortical TUNEL‐positive cells induced by miR‐29b antagomir in MCAO rats (Figure 4B, *p* < 0.05). C1QTNF6 mRNA and protein in the leukocytes and brain were significantly higher in MCAO rats treated with miR‐29b antagomir than in MCAO rats. However, this increase was downregulated by H19 siRNA treatment (Figure 4C,D; *p *< 0.05). Cerebral and leukocyte TNF‐α and IL‐1β levels were decreased in rats treated with H19 siRNA as compared to that in MCAO rats treated with miR‐29b antagomir (Figure 4C,D; *p *< 0.05). These findings suggest that H19 promotes TNF‐α and IL‐1β secretion by targeting the miR‐29b/C1QTNF6 axis in leukocytes.

### 3.7 H19 and miR‐29b in HL‐60 cells regulate hCMEC/D3 apoptosis

**FIGURE 5:**
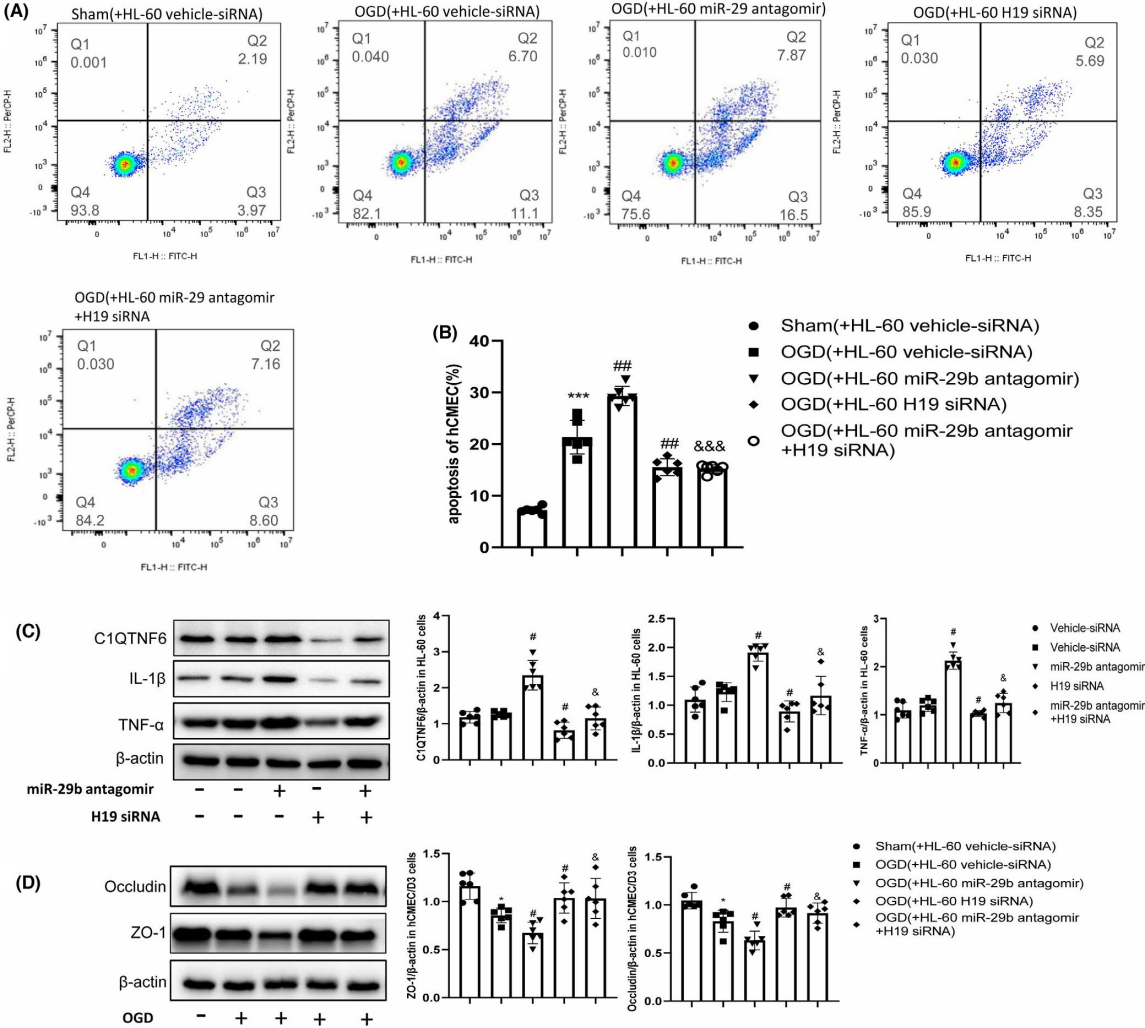
H19 and miR‐29b regulate ZO‐1 and occludin expression in hCMEC/D3 cells. (A and B) hCMEC/D3 cell apoptosis induced by H19 siRNA and miR‐29b antagomir treatment (*N* = 6). (C) C1QTNF6, IL‐1β, and TNF‐α protein levels in HL‐60 cells assessed by Western blotting (*N* = 6). (D) ZO‐1 and occludin protein levels in hCMEC/D3 cells assessed by Western blotting (*N* = 6). *p < 0.05 vs. sham group; ***p < 0.001 vs. sham group; ^#^ *p* < 0.05 vs. OGD+vehicle‐siRNA group or vehicle‐siRNA group; ^##^ *p* < 0.01 vs. OGD+vehicle‐siRNA group or vehicle‐siRNA group; ^&^ *p* < 0.05 vs. OGD+miR‐29b antagomir group; ^&&&^ *p* < 0.001 vs. OGD+miR‐29b antagomir group

To explore whether leukocyte C1QTNF6 affects hypoxia‐induced BBB disruption through an inflammatory response, we established an in vitro model in which OGD‐exposed hCMEC/D3 cells were co‐cultured with HL‐60 cells. HL‐60 cells in different groups were treated as described previously. Flow cytometry analysis revealed a decreased apoptosis ratio in hCMEC/D3 cells in the OGD (+HL‐60 H19 siRNA) group than in the OGD (+HL‐60 vehicle‐siRNA) group (Figure 5A,B; *p* < 0.05). However, there was an increased apoptosis ratio in hCMEC/D3 cells in the OGD (+HL‐60 miR‐29b antagomir) group than in the OGD (+HL‐60 vehicle‐siRNA) group (Figure 5A,B; *p* < 0.05). Increased hCMEC/D3 cell apoptosis induced by OGD and HL‐60 miR‐29b antagomir was reversed by HL‐60 H19 siRNA treatment (Figure 5A,B; *p* < 0.05).

Additionally, we measured C1QTNF6, IL‐1β, and TNF‐α expression levels in HL‐60 cells, and ZO‐1 and occludin expression levels in hCMEC/D3 cells. Treatment with miR‐29b antagomir significantly increased the expression of C1QTNF6, IL‐1β, and TNF‐α in HL‐60 cells (Figure 5C, *p* < 0.05). There was a significant decrease in the expression levels of C1QTNF6, IL‐1β, and TNF‐α in HL‐60 cells treated with H19 siRNA as compared to that in OGD HL‐60 cells (Figure 5C, *p* < 0.05). Furthermore, we observed changes in ZO‐1 and occludin levels in hCMEC/D3 cells and changes in C1QTNF6, IL‐1β, and TNF‐α levels in HL‐60 cells. (Figure 5D). These findings suggest that H19 in leukocytes aggravates hypoxia‐induced hCMEC/D3 apoptosis by targeting the miR‐29b/C1QTNF6 axis.

## 4 DISCUSSION

Our findings confirmed the upregulation of H19 in neutrophils of patients with acute ischemic stroke, which is positively associated with the severity of neurological deficits. Moreover, we observed miR‐29b downregulation in the neutrophils of ischemic stroke patients, which was negatively associated with H19 levels. Additionally, we demonstrated that H19 regulates C1QTNF6 expression by sponging miR‐29b, which facilitates the release of IL‐1β and TNF‐α by leukocytes and BBB disruption during cerebral ischemic injury. Our findings provide new insights for possible targeted treatments of ischemic stroke.

Emerging evidence regarding peripheral immune cells has provided insights into novel inflammatory mechanisms contributing to neuronal cell death in cerebral ischemic injury.^16^, ^17^ Leukocytes are sensitive indicators of inflammatory stimuli in patients with ischemic stroke.^18^, ^19^ There is altered leukocyte expression of multiple ncRNAs in patients with ischemic stroke, including H19, which is crucially involved in the inflammatory response of ischemic stroke.^13^ We previously reported that H19 increases the immune response by increasing plasma TNF‐α and IL‐1β levels after ischemic stroke; moreover, it affects the subsequent pathological outcome.^13^ Wan et al. found that H19 upregulates MCAO‐induced IL‐1β and IL‐18 expression by triggering caspase‐1 signals; moreover, H19 excision exerts anti‐inflammatory effects by inhibiting the production of these cytokines.^20^ Additionally, H19 functions as a competitive endogenous RNA for regulating neuronal apoptosis by sponging different miRNAs. Cheng et al. showed the upregulation of H19 in patients with diabetes mellitus, which directly suppresses miR‐29b levels.^21^ A recent study reported that H19 inhibition ameliorated OGD‐induced hippocampal neuronal apoptosis and increased inflammatory cytokine levels by directly targeting miR‐29b.^22^ We observed significant upregulation of H19 in leukocytes of patients with acute ischemic stroke and MCAO rats; moreover, H19 excision suppressed IL‐1β and TNF‐α secretion by directly targeting miR‐29b. In addition, we identified the binding site of miR‐29b in H19 using the StarBase database. Our findings suggest that H19 and miR‐29b interactions are crucially involved in inflammatory signaling during cerebral ischemic injury.

Multiple studies have demonstrated that miR‐29b is a key mediator of ischemic cascade in regulating inflammation and neuronal apoptosis.^23^ There is a decreased miR‐29b expression in ischemic regions and OGD‐activated microglia, which contributes to injury in focally ischemic brains.^24^ Additionally, miR‐29b can inhibit neuronal apoptosis by suppressing TNF‐α and IL‐1β release in OGD‐activated microglia.^24^ The miR‐29b level in the blood of patients significantly decreased with ischemic stroke and in the blood and brain of patients with cerebral ischemic injury.^12^ This suggests that miR‐29b in the brain and circulating blood could be involved in regulating the post‐stroke immune response, which is consistent with our findings. We found that miR‐29b excision promoted neuronal apoptosis and upregulated TNF‐α and IL‐1β expression by targeting C1QTNF6. Similarly, Wang et al. reported that miR‐29b promoted IL‐1β, IL‐6, and IL‐8 expression by inhibiting C1QTNF6 in human bronchial epithelial cells.^25^

It was initially found that C1QTNF6 exerts regulates inflammatory response in several diseases.^26^ C1QTNF6 is crucially involved in alleviating OGD‐induced inflammatory molecules, including TNF‐α and IL‐1β in PC12 cells.^27^ C1QTNF6 induces IL‐10 expression in macrophages, which may present a novel target for pharmacological therapies in inflammatory diseases.^28^ Consistent with the previous findings, we found that C1QTNF6 is among the target genes of miR‐29b, and is significantly downregulated in leukocytes of stroke patients and MCAO rats. However, C1QTNF6 overexpression with miR‐29b inhibition promoted MCAO‐induced expression of pro‐inflammatory cytokines in leukocytes. Peripheral inflammatory factors, including IL‐1β and TNF‐α, are more likely to aggravate BBB damage and even worsen stroke outcomes.^29^ IL‐1β and TNF‐α could reduce the expression of tight junction proteins or lead to the formation of false adjacent blood vessels and further destroy BBB integrity and normal function.^30^ Neutrophils produce various pro‐inflammatory cytokines that affect BBB function, including IL‐1β and TNF‐α, which promotes neutrophil recruitment to the brain parenchyma and exacerbates inflammation.^31^ We observed increased IL‐1β and TNF‐α secretion after C1QTNF6 overexpression in the peripheral leukocytes of MCAO rats. Additionally, increased IL‐1β and TNF‐α levels in the brain promote leukocyte migration into the brain parenchyma and increase BBB permeability, which is a vicious cycle. Consistent with our *in vivo* study, C1QTNF6 overexpression promoted IL‐1β and TNF‐α expression in HL‐60 cells. IL‐1β and TNF‐α upregulation can directly cause apoptosis of endothelial cells, which are important components of BBB.

## 5 CONCLUSIONS

In summary, this is the first study to demonstrate H19 and C1QTNF6 upregulation and miR‐29b downregulation in leukocytes of patients with ischemic stroke and MCAO rats. H19 promotes C1QTNF6 expression by directly targeting miR‐29b in leukocytes of MCAO rats. Furthermore, C1QTNF6 upregulation in leukocytes aggravates cerebral ischemic injury and promotes BBB damage through IL‐1β and TNF‐α upregulation. Our findings demonstrate the inflammation‐modulatory effect of the H19/miR‐29b/C1QTNF6 axis in the mechanism of cerebral ischemia injury.

## CONFLICTS OF INTEREST

The authors declare that they have no competing interests.

## AUTHOR CONTRIBUTIONS

G.L. and X.M. conducted the study design and animal and histology experiments, and prepared the manuscript. H.Z., J.F., and T.L. designed the clinical experiment. Y.L. and Y.G. designed and supervised the research.

## Data Availability

The datasets used and analyzed during the current study are available from the corresponding author on reasonable request.
